# Working together to ensure the realisation of ‘life to years’

**DOI:** 10.1007/s12471-022-01739-y

**Published:** 2022-11-14

**Authors:** R. Collins

**Affiliations:** 1 Geriatric and Stroke Medicine, Tallaght University Hospital, Tallaght, Dublin, Ireland; 2 Department of Gerontology, Trinity College Dublin, Dublin, Ireland

The paper by Boerlage-van Dijk et al. from the Dutch Working Group of Geriatric Cardiology in this issue of the *Netherlands Heart Journal* considers what is missing from European guidelines regarding the management of cardiovascular disease in older people [1]. This continues a momentum of a European-wide focus on the health of older people as we acknowledge the challenge of our changing demography. It was previously estimated, for example, that by 2060, the number of adults over 55 years with atrial fibrillation (AF) would account for 3.2% of the Dutch population and that this would be mainly due to a trebling of the prevalence in the over-75s [2]. While we must celebrate the success of medicine in achieving longevity and seek to fully reap the harvest it also brings to society, this cannot be achieved without preparing to ensure health in later life and adding ‘life to years’ too.

In their important ‘Point of View’, Boerlage-van Dijk et al. rightly acknowledge that although cardiovascular disease is most prevalent in an older and frailer population, much of our research for its treatment has not included these cohorts of patients and our evidence base is both limited and myopic in its lack of consideration of older people. The authors highlight the fact that while most European Society of Cardiology (ESC) guidelines mention ‘frailty’ and ‘shared decision making’, the details of actualising such assessment and procedure are somewhat ignored. Expanding on topics such as frailty, age-appropriate prescribing, advance care planning etc. may be felt to be beyond the scope of a cardiovascular guideline, but even in that insinuation, there is a myopia to the needs of older people for whom cardiovascular disease may be just one of several comorbidities and health concerns that are also important determinants of these patients’ function and independence.

The authors correctly identify the need for other skills and knowledge bases such as in palliative care that are required in this arena of medicine. Of the 27 guidelines identified, just about half have a dedicated subchapter on older people and only a third define what ‘an older patient’ is, which is usually the outdated concept of a retirement age of 65 years. It is noteworthy that although advocating for a holistic integrated model of care for older patients with AF, the ESC in its illustration of the ideal continuum of care singularly omitted the specialty of geriatric medicine/medical gerontology. This points to an agnosia of important and effective skills this specialty has to contribute through its comprehensive geriatric assessment, model of care [3, 4].

This important principle of comprehensive assessment and the potential for multidisciplinary value-added input in the health of older people with cardiovascular disease has been recognised by the Horizon 2020 EHRA-PATHS project, which seeks to improve the care of older people with AF. Funded by the European Union Horizon 2020 Fund, it will devise a software-based holistic care plan for older people with AF that is cognisant of other cardiovascular issues such as heart failure, hypertension and stroke, as well as other comorbidities such as polypharmacy, acute trauma, cancer and dementia. It will hopefully prove itself to be the model of best practice for all future cardiovascular care in a planned randomised controlled trial [5].

It is laudable that there is a Dutch geriatric cardiology working group, and there have been similar encouraging developments in other areas of medicine, such as geriatric oncology. Perhaps it is time to recognise that older people make up a majority of many medical disease classifications and that greater subspecialty training and interdisciplinary collaboration between those who specialise in such diseases and those who are specialist in the care of older people can only result in better models of care for patients and health services. As Boerlage-van Dijk and colleagues have illustrated, this paradigm is currently not often reflected in our clinical guidelines.
